# Asian Genotype Zika Virus Detected in Traveler Returning to Mexico from Colombia, October 2015

**DOI:** 10.3201/eid2205.160190

**Published:** 2016-05

**Authors:** José Alberto Díaz-Quiñonez, Noé Escobar-Escamilla, Claudia Wong-Arámbula, Mauricio Vázquez-Pichardo, Belem Torres-Longoria, Irma López-Martínez, Cuitláhuac Ruiz-Matus, Pablo Kuri-Morales, José Ernesto Ramírez-González

**Affiliations:** Universidad Nacional Autónoma de México Facultad de Medicina, Mexico City, Mexico (J.A. Díaz-Quiñonez);; Instituto de Diagnóstico y Referencia Epidemiológicos “Dr. Manuel Martínez Báez,” Mexico City (J.A. Díaz-Quiñonez, N. Escobar-Escamilla, C. Wong-Arámbula, M. Vázquez-Pichardo, B. Torres-Longoria, I. López-Martínez, J.E. Ramírez-González);; Dirección General de Epidemiología, Mexico City (C. Ruiz-Matus);; Subsecretaría de Prevención y Promoción de la Salud, Mexico City (P. Kuri-Morales)

**Keywords:** Zika virus, Asian genotype, travel, Mexico, Colombia, viruses

**To the Editor:** Zika virus is an emerging arbovirus spread by *Aedes aegypti* mosquitoes and belongs to the genus *Flavivirus* of the Spondweni serocomplex (*1*,*2*). Most often, signs and symptoms of infection are maculopapular rash, fever, arthralgia, myalgia, headache, and conjunctivitis; edema, sore throat, cough, and vomiting occur less frequently. 

Zika virus is an RNA virus containing 10,794 nt, and diagnostic tests include PCRs on acute-phase serum samples to detect viral RNA (*1*). The genome contains 5′ and 3′ untranslated regions flanking a single open reading frame (ORF) that encodes a polyprotein that is cleaved into the structural proteins capsid (C), premembrane/membrane (prM), and envelope (E), and 8 non-structural proteins (NS1, NS2A, NS2B, NS3, NS4A, 2K, NS4B, and NS5) (*3*). Genetic studies in which nucleotide sequences derived from the NS5 gene were used indicated 3 Zika virus lineages: East African, West African, and Asian (*4*,*5*). 

In Brazil, the first identified cases of dengue-like syndrome with subsequent Zika virus confirmation were documented in the early months of 2015 in the state of Rio Grande do Norte (*6*). Later that year, autochthonous transmission was reported in Colombia and Suriname during October–November (*5*) and Puerto Rico in December (*6*,*7*). During the same period, imported cases in the United States and Mexico were reported (*6*). By December 2015, we had already identified at least 15 autochthonous and 1 imported Zika cases in Mexico, initially detected by real-time reverse transcription PCR (RT-PCR). Here, we report on the documentation of a case of Zika virus infection in a male traveler returning to Mexico from Colombia in October 2015. 

On October 21, 2015, we identified an imported case of Zika virus infection in the central state of Querétaro, Mexico. The patient, a 26-year-old man, had visited Santa Martha, Colombia, during the previous 12 days. Symptoms including fever, muscle pain, mild to moderate arthralgia, arthritis, back pain, chills, and conjunctivitis began on October 19, two days after his return to Mexico. A sample was collected at a primary healthcare clinic. Initial molecular testing for dengue virus at the Queretaro Public Health Laboratory was negative; to test for Zika virus, the sample was sent to the National Reference Laboratory (InDRE), where viral RNA was extracted from it by using the QIAamp Viral RNA Mini Kit (QIAGEN, Hilden, Germany). We used real-time RT-PCR for diagnosis, using the Superscript III system (Invitrogen, Carlsbad, CA, USA) and primers and probes previously reported (*8*). Using Zika virus nucleotide sequence data in the Primer3Plus web interface (*8*), we amplified a 760-bp fragment with the following primers for partial characterization of viral NS5 coding gene: ZikV9113Fwd TTYGAAGCCCTTGGATTCTT and ZikV9872Rev CYCGGCCAATCAGTTCATC. We used the QIAGEN One-Step RT-PCR Kit as follows: reverse transcription at 50°C for 30 min, followed by an activation step at 95°C for 15 min and 35 cycles of 94°C for 30 sec, 55°C for 30 sec, and 72°C for 1 min, and a final extension step at 72°C for 10 min. We sequenced amplicons in the ABI PRISM 3130xl Genetic Analyzer instrument using the BigDye Terminator v3.1 Cycle Sequencing kit (Applied Biosystems, Foster City, CA). The partial sequence of the identified strain ((MEX/InDRE/14/2015) was deposited in GenBank under accession no. KU556802. 

We performed phylogenetic analysis to compare the extracted sequences with a database of 39 available nucleotide sequences from GenBank (Figure). Sequences from NS5 data were aligned, the dataset was adjusted to a common size of 531 pb, and a phylogenetic tree was constructed in MEGA6 (http://www.megasoftware.net) from aligned nucleotide sequences. The maximum-likelihood statistical algorithm and the Tamura-Nei substitution model with 1,000 replicates for bootstrap were used. Phylogenetic analyses showed that the partially sequenced strain MEX/InDRE/14/2015 belongs to the Zika virus Asian lineage and is closely related to those reported from Brazil and Suriname in 2015 (Figure). The phylogeny does show some genetic distance with respect to strains causing outbreaks in 2014 in the Americas, suggesting acquired genetic changes probably caused by adaptations during the spread of the virus, similar to those observed for chikungunya virus (*9*).

**Figure:**
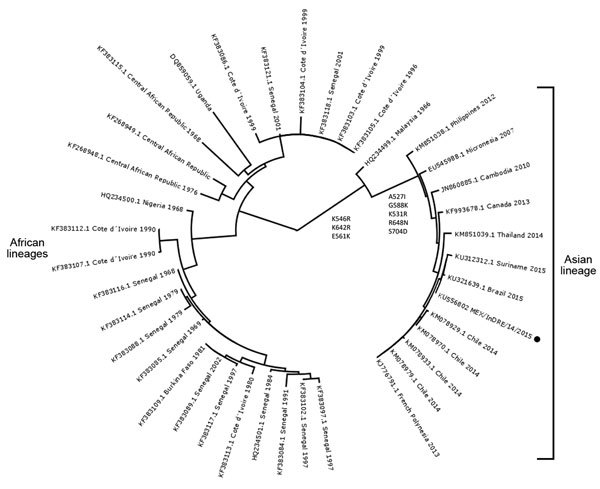
Phylogenetic analysis of nonsegmented protein 5 partial sequences of Zika virus isolated from a traveler returning from Colombia to Mexico (MEX/InDRE/14/2015; black dot), October 2015, showing close relationship Zika virus strains reported from Brazil and Suriname in 2015. We determined the evolutionary relationship implementing the maximum likelihood statistical algorithm and the Tamura-Nei substitution model using MEGA6 (http://www.megasoftware.net). The tree was created by using FigTree version 1.4.2 software (http://tree.bio.ed.ac.uk/software/figtree/). Molecular markers are indicated near the node source. Strain labels consist of GenBank accession number, country, and year of isolation.

We conducted a nonsynonymous mutation analysis using the NS5 protein from the Zika virus isolated in French Polynesia in 2013 (903 aa; GenBank accession no. KJ776791.1) as a reference. The strain MEX/InDRE/14/2015bears the mutation markers K546R, K642R, and E561K, which cause the differentiation of the Asian lineage from the clades representing the African lineage (Figure). In addition, we observed that markers A527I, G588K, K531R, R648N, and S704D were acquired during the virus dispersion from Southeast Asia to the Pacific region and the Americas.

In summary, we identified Zika virus in a traveler who returned from Colombia to Mexico in October 2015. A partial sequence of the NS5 gene showed that the isolate from this patient was closely related to those described elsewhere in the Western Hemisphere belonging to the Asian lineage, particularly to 2 strains identified in Brazil and Suriname during 2015.
